# Clinical Relevance of Local Renin Angiotensin Systems

**DOI:** 10.3389/fendo.2014.00113

**Published:** 2014-07-14

**Authors:** Duncan J. Campbell

**Affiliations:** ^1^St. Vincent’s Institute of Medical Research, Fitzroy, VIC, Australia; ^2^Department of Medicine, University of Melbourne, St. Vincent’s Hospital, Fitzroy, VIC, Australia

**Keywords:** renin, angiotensin, angiotensinogen, angiotensin converting enzyme, chymase

The concept of a “local” renin angiotensin system (RAS) can mean different things to different people. Its main purpose is to differentiate the “local” RAS operating in tissues from the classical “circulating” RAS, but it is difficult to differentiate between the two systems because of their extensive overlap. The circulating RAS comprises kidney-derived renin acting on liver-derived angiotensinogen to generate angiotensin (Ang) I that is converted to Ang II by angiotensin converting enzyme (ACE). However, tissues are the main site of production of angiotensin peptides by the circulating RAS, whereby plasma-derived renin acts on plasma-derived angiotensinogen to generate Ang I, which is converted to Ang II by endothelial ACE (1–4).

Local RAS refers to tissue-based mechanisms of Ang peptide formation that operate separately from the circulating RAS. Although many different concepts of local RAS have been described, a key feature is the local synthesis of RAS components including angiotensinogen and enzymes such as renin that cleave angiotensinogen to produce Ang peptides independently of the circulating RAS. ACE and Ang II type 1 (AT1) and type 2 (AT2) receptors are invariably locally synthesized, but these are also components of the circulating RAS. Many other potential components of local RAS have been described that may contribute to tissue-specific mechanisms of Ang peptide formation, and that may either participate in disease processes or contribute to mechanisms that protect from tissue injury. These include the (pro)renin receptor (5), renin-independent mechanisms of Ang peptide generation from Ang- (1-12) (6), intracellular (or intracrine) RAS that may contribute to cardiovascular disease (7, 8), and AT2 receptors (7) and the ACE2/Ang-(1-7)/Mas receptor pathway (6–8) that may mediate therapeutic benefit in cardiovascular disease. In addition, novel Ang peptides with novel pharmacology, including Ang IV, Ang A, alamandine, and angioprotectin (6, 8), have the potential to contribute to disease or to protective mechanisms. Moreover, the brain RAS, including the ACE2/Ang-(1-7)/Mas receptor and the Ang IV/insulin regulated aminopeptidase pathways may play a role in Alzheimer’s and Parkinson’s diseases (9). Local production of aldosterone may have a pathogenic role (7, 10), ACE, AT2 receptors, Ang-(1-7) and acetyl-Ser-Asp-Lys-Pro may have a role in hematopoiesis (11), and the ACE2/Ang-(1-7)/Mas receptor pathway may contribute to fetal programing, reproduction, and cancer (6, 12).

This short opinion piece discusses the potential clinical relevance of local RAS. The challenge in demonstrating the independence of local from circulating RAS, and the potential interaction of ACE inhibitor and AT1 receptor blocker (ARB) therapies with local RAS are discussed. Attempts to define local RAS that are independent of the circulating RAS have been primarily based on animal models and the clinical relevance of local RAS is uncertain. However, this area of research continues to evolve, and today’s opinions may change as we gain better understanding of how these novel components and mechanisms impact on clinical medicine.

## How Can Local RAS be Shown to be Independent of the Circulating RAS?

As reviewed elsewhere (5–12), many lines of evidence suggest the possibility of local RAS that may operate independently of the circulating RAS and play a pathogenic or protective role. This evidence includes the widespread tissue expression of angiotensinogen, the only known precursor of the Ang peptides and an essential requirement for an independent local RAS (13–16). However, local production of RAS components does not prove their functional significance, and proving their clinical relevance presents many challenges. One approach to study of the role of locally synthesized RAS components is their targeted deletion from specific tissues. This approach has been applied to the kidney.

Both clinical experiences with ACE inhibitor and ARB therapies during pregnancy, and ACE, renin, angiotensinogen, and AT1 receptor gene mutation and knockout models demonstrate a critical role for the RAS in renal development and function in animals and humans (17–23). Moreover, ACE inhibition demonstrates a differential regulation of Ang II levels in kidney and blood (24). However, these data do not prove a specific role for the local RAS in the kidney. Matsusaka et al. investigated the role of the local RAS in renal development and function by producing mice with genetic deletion of angiotensinogen synthesis in the kidney. In contrast to the morphological and functional consequences of whole body or liver specific deletion of angiotensinogen gene expression, deletion of angiotensinogen production in the kidney had no effect on renal morphology or function (25). Moreover, contrary to the expectation that locally produced angiotensinogen was the main contributor to renal Ang II levels, Matsusaka et al. showed deletion of renal angiotensinogen production had no effect on renal Ang II levels, and that liver angiotensinogen is the primary source of Ang II in the kidney (25). With the caveat that the studies of Matsusaka et al. were not in pathophysiological models (25), these data show that evidence for local synthesis of a RAS component is not sufficient to establish a role for the locally synthesized component in physiology or pathology, whether by an intracellular (intracrine) or extracellular mechanism. Proof that a locally synthesized RAS component contributes to physiology or pathology requires demonstration that deletion of the locally synthesized component impacts on physiology and/or pathology.

Similar to the case for angiotensinogen, mice with reduced renal expression of ACE had normal histology and urine concentrating ability (26), suggesting that locally synthesized ACE does not play an essential role in normal renal development and function. Moreover, the marked reduction in Ang II levels in kidney, heart, and other organs caused by global ACE gene deletion, despite near-normal Ang I levels (27, 28), indicates that an intracellular (intracrine) ACE-independent mechanism of Ang II formation is unlikely to exist in these tissues.

Evidence for a pathogenic role of renal ACE is the demonstration that genetic deletion of renal ACE expression prevented hypertension produced by subcutaneous administration of Ang II (26), suggesting a specific renal ACE-dependent mechanism of hypertension in this model. However, the significance of this finding is uncertain because ACE inhibition does not modify hypertension produced by intravenous Ang II administration in either animal of human studies (29–33), and it is questionable whether the subcutaneous Ang II model of hypertension has any physiological or pathological relevance (34).

An alternative approach to defining a local tissue RAS was to use recombinant technology to express ACE as a reporter gene on the cardiomyocyte membrane (35). In this model, ACE expression on the cardiomyocyte membrane (where it is not normally expressed) would be expected to increase cardiac Ang II levels if Ang I were also present in this tissue compartment. Expression of ACE on the cardiomyocyte membrane increased cardiac Ang II levels in mice without endothelial expression of ACE, but not in rats or mice with endothelial ACE expression (35, 36). These studies do not therefore provide evidence in support of Ang I formation in the extravascular compartment of the heart of animals with endothelial ACE expression. By contrast, deletion of testicular ACE reduced male fertility (37), indicating a specific role for testicular ACE. However, ACE has many substrates (38) and the reduction in male fertility may reflect an action of testicular ACE that is independent of Ang peptides.

Part of the challenge in identifying a local RAS that is independent of the circulating RAS is the difficulty in measuring *in vivo* levels of Ang peptides in tissues. For example, initial reports of substantial amounts of Ang II and Ang-(1-7) in the brain (39, 40) were not confirmed when more rigorous methodology was applied (41, 42).

## Do the Therapeutic Benefits of ACE Inhibitor and ARB Therapies Establish the Clinical Relevance of Local RAS?

A key argument in support of the clinical relevance of the RAS, whether local or circulating, is the therapeutic benefit from inhibition of this system. De Mello and Frohlich proposed that the local RAS mediates in part the therapeutic benefits of ACE inhibitor and ARB therapies (7), but there are difficulties in establishing such a role for local RAS. For example, the claim that the beneficial effects of these therapies occurred independently of blood pressure (7) suggests, but does not prove, a role for local RAS. The complexity of blood pressure regulation means that alternative explanations are possible and ambulatory blood pressure monitoring may be necessary to demonstrate an effect of therapy on blood pressure not detected by office blood pressure measurement (43). Furthermore, the different benefits of ACE inhibitor and ARB therapies in comparison with antihypertensive agents that act independently of the RAS (7) do not prove that these benefits were due to inhibition of local rather than the circulating RAS.

**Figure 1:**
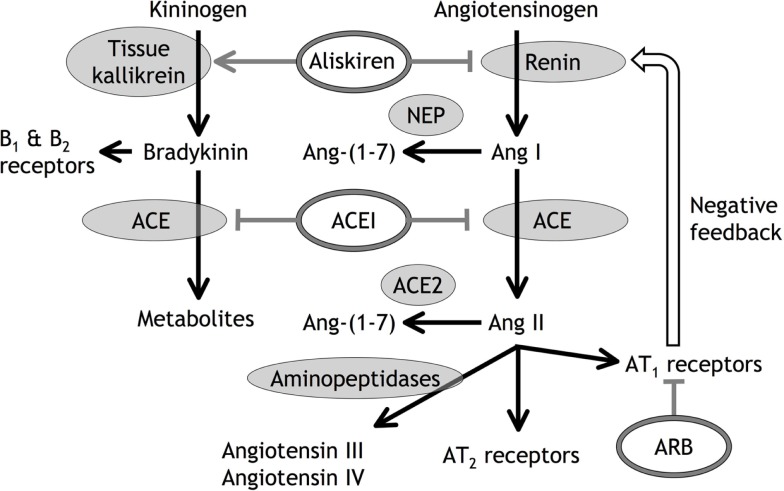
**Diagrammatic representation of angiotensin (Ang) and bradykinin peptide formation and metabolism, with the sites of action of angiotensin converting enzyme (ACE) inhibitors (ACEI), angiotensin type 1 (AT_1_) receptor blockers (ARB), and the renin inhibitor aliskiren**. In addition to inhibiting renin, aliskiren increases tissue kallikrein activity and bradykinin levels that may act on bradykinin type 1 (B_1_) and type 2 (B_2_) receptors (55). Neutral endopeptidase (NEP) converts Ang I to Ang-(1-7), ACE2 converts Ang II to Ang-(1-7), and aminopeptidases convert Ang II to Ang III and Ang IV.

Ang II administration is a well-recognized model of cardiovascular and renal disease (44–46), and the therapeutic benefits of RAS inhibition are almost certainly in large part a consequence of reduced Ang II stimulation of the AT1 receptor in high renin, high Ang II conditions such as renal artery stenosis and heart failure. Reduced AT1 receptor stimulation may also play an important role in the renal effects of RAS inhibition, including the side effects of these therapies (47, 48). Many studies investigating the combination of ACE inhibitor, ARB, and renin inhibitor therapies were based on the assumption that the therapeutic benefits of these agents are the consequence of reduced AT1 receptor stimulation, and that combination of these therapies would produce greater therapeutic benefit by producing greater reduction in AT1 receptor stimulation (47–53). What may not have been appreciated was the large body of preclinical and clinical data indicating that these drugs also produce benefits by mechanisms separate from reduced AT1 receptor stimulation. Moreover, many of these mechanisms separate from reduced AT1 receptor stimulation involve novel RAS components implicated in local tissue RAS (Figure 1). For example, ARB therapies, by blocking the negative feedback control of renin secretion, also increase Ang II levels that stimulate the AT2 receptor, leading to cardioprotection (54, 55). Moreover, both ACE inhibitor and ARB therapies increased Ang-(1-7) levels (56) that may produce therapeutic effects mediated by the Ang-(1-7)/Mas receptor pathway (6). In addition, ACE inhibitor, ARB, and renin inhibitor therapies increase bradykinin levels that may contribute to their antihypertensive and cardioprotective actions (54, 55, 57–63). Consequently, therapeutic benefit from ACE inhibitor, ARB, and renin inhibitor therapies does not prove a pathogenic role for the RAS, either local or circulating.

An important aspect of these additional mechanisms of therapeutic benefit from RAS inhibition is that combination of ACE inhibitor, ARB, and/or renin inhibitor therapies may block some of these mechanisms of benefit, thereby explaining the many clinical studies, apart from heart failure (49), that showed no additional benefit from combination of ACE inhibitor, ARB, and renin inhibitor therapies (47, 48, 50–53). For example, the benefits of ARB therapy produced by increased Ang II levels and AT2 receptor stimulation will be blocked if combined with renin inhibitor or ACE inhibitor therapies, because renin inhibitor and ACE inhibitor therapies attenuate the increase in Ang II levels produced by ARB therapy (55, 56, 64, 65). Moreover, the benefits of ACE inhibitor and ARB therapies produced by increased Ang-(1-7) levels and Mas receptor stimulation will be blocked if combined with renin inhibitor or neutral endopeptidase inhibitor therapies because renin inhibitor and neutral endopeptidase inhibitor therapies attenuate the increase in Ang-(1-7) levels produced by ACE inhibitor and ARB therapies (66). In addition, neutral endopeptidase inhibitor therapy may increase Ang II levels by reducing Ang II metabolism (66, 67).

## Conclusion

Current concepts of the local RAS have expanded to include the (pro)renin receptor, renin-independent mechanisms of Ang peptide generation from Ang-(1-12), AT2 receptors, the ACE2/Ang-(1-7)/Mas receptor and Ang IV/insulin regulated aminopeptidase pathways, an intracellular (intracrine) RAS, and novel Ang peptides (5–9, 11, 12). Much of the evidence for these new RAS components is based on animal studies and further research is required to establish that local RAS contribute to physiology and disease. Consequently, the clinical relevance of local RAS remains speculative. Nevertheless, the expanding repertoire of local RAS components offers new therapeutic targets and the prospect of new therapies.

## Conflict of Interest Statement

The author declares that the research was conducted in the absence of any commercial or financial relationships that could be construed as a potential conflict of interest.

## References

[1] Campbell DJ The site of angiotensin production. J Hypertens (1985) 3:199–207 10.1097/00004872-198506000-00002 3894514

[2] Campbell DJ Circulating and tissue angiotensin systems. J Clin Invest (1987) 79:1–6 10.1172/JCI112768 3025255 PMC423969

[3] Admiraal PJJ Derkx FHM Danser AHJ Pieterman H Schalekamp MADH Metabolism and production of angiotensin I in different vascular beds in subjects with hypertension. Hypertension (1990) 15:44–55 10.1161/01.HYP.15.1.44 2403979

[4] Campbell DJ Angiotensin II generation in vivo: does it involve enzymes other than renin and angiotensin-converting enzyme? J Renin Angiotensin Aldosterone Syst (2012) 13:314–6 10.1177/1470320312447162 22626976

[5] Binger KJ Muller DN Autophagy and the (pro)renin receptor. Front Endocrinol (2013) 4:155 10.3389/fendo.2013.00155 PMC3800846 24155743

[6] Chappell MC Marshall AC Alzayadneh EM Shaltout HA Diz DI Update on the angiotensin converting enzyme 2-angiotensin (1-7)-Mas receptor axis: fetal programing, sex differences, and intracellular pathways. Front Endocrinol (2014) 4:201 10.3389/fendo.2013.00201 24409169 PMC3886117

[7] De Mello WC Frohlich ED Clinical perspectives and fundamental aspects of local cardiovascular and renal renin-angiotensin systems. Front Endocrinol (2014) 5:16 10.3389/fendo.2014.00016 24600438 PMC3928588

[8] Zhuo JL Ferrao FM Zheng Y Li XC New frontiers in the intrarenal renin-angiotensin system: a critical review of classical and new paradigms. Front Endocrinol (2013) 4:166 10.3389/fendo.2013.00166 24273531 PMC3822323

[9] Wright JW Kawas LH Harding JW A role for the brain RAS in Alzheimer’s and Parkinson’s diseases. Front Endocrinol (2013) 4:158 10.3389/fendo.2013.00158 PMC3829467 24298267

[10] Aroor AR Demarco VG Jia G Sun Z Nistala R Meininger GA The role of tissue renin-angiotensin-aldosterone system in the development of endothelial dysfunction and arterial stiffness. Front Endocrinol (2013) 4:161 10.3389/fendo.2013.00161 24194732 PMC3810594

[11] Rodgers KE Dizerega GS Contribution of the local RAS to hematopoietic function: a novel therapeutic target. Front Endocrinol (2013) 4:157 10.3389/fendo.2013.00157 24167502 PMC3805949

[12] Herr D Bekes I Wulff C Local renin-angiotensin system in the reproductive system. Front Endocrinol (2013) 4:150 10.3389/fendo.2013.00150 PMC3798827 24151488

[13] Campbell DJ Habener JF Angiotensinogen gene is expressed and differentially regulated in multiple tissues of the rat. J Clin Invest (1986) 78:31–9 10.1172/JCI112566 3013940 PMC329527

[14] Campbell DJ Habener JF Cellular localization of angiotensinogen gene expression in brown adipose tissue and mesentery: quantification of messenger ribonucleic acid abundance using hybridization *in situ*. Endocrinology (1987) 121:1616–26 10.1210/endo-121-5-1616 3665835

[15] Campbell DJ Habener JF Hybridization *in situ* studies of angiotensinogen gene expression in rat adrenal and lung. Endocrinology (1989) 124:218–22 10.1210/endo-124-1-218 2909366

[16] Campbell DJ Sernia C Thomas WG Oldfield BJ Immunocytochemical localization of angiotensinogen in rat brain: dependence of neuronal immunoreactivity on method of tissue processing. J Neuroendocrinol (1991) 3:653–60 10.1111/j.1365-2826.1991.tb00330.x 19215535

[17] Bullo M Tschumi S Bucher BS Bianchetti MG Simonetti GD Pregnancy outcome following exposure to angiotensin-converting enzyme inhibitors or angiotensin receptor antagonists: a systematic review. Hypertension (2012) 60:444–50 10.1161/HYPERTENSIONAHA.112.196352 22753220

[18] Moreno C Hoffman M Stodola TJ Didier DN Lazar J Geurts AM Creation and characterization of a renin knockout rat. Hypertension (2011) 57:614–9 10.1161/HYPERTENSIONAHA.110.163840 21242461 PMC3513323

[19] Yanai K Saito T Kakinuma Y Kon Y Hirota K Taniguchi-Yanai K Renin-dependent cardiovascular functions and renin-independent blood- brain barrier functions revealed by renin-deficient mice. J Biol Chem (2000) 275:5–8 10.1074/jbc.275.1.5 10617578

[20] Gribouval O Moriniere V Pawtowski A Arrondel C Sallinen SL Saloranta C Spectrum of mutations in the renin-angiotensin system genes in autosomal recessive renal tubular dysgenesis. Hum Mutat (2012) 33:316–26 10.1002/humu.21661 22095942

[21] Niimura F Labosky PA Kakuchi J Okubo S Yoshida H Oikawa T Gene targeting in mice reveals a requirement for angiotensin in the development and maintenance of kidney morphology and growth factor regulation. J Clin Invest (1995) 96:2947–54 10.1172/JCI118366 8675666 PMC186006

[22] Tsuchida S Matsusaka T Chen XM Okubo S Niimura F Nishimura H Murine double nullizygotes of the angiotensin type 1A and 1B receptor genes duplicate severe abnormal phenotypes of angiotensinogen nullizygotes. J Clin Invest (1998) 101:755–60 10.1172/JCI1899 9466969 PMC508622

[23] Esther CR Jr. Howard TE Marino EM Goddard JM Capecchi MR Bernstein KE Mice lacking angiotensin-converting enzyme have low blood pressure, renal pathology, and reduced male fertility. Lab Invest (1996) 74:953–65 8642790

[24] Campbell DJ Lawrence AC Towrie A Kladis A Valentijn AJ Differential regulation of angiotensin peptide levels in plasma and kidney of the rat. Hypertension (1991) 18:763–73 10.1161/01.HYP.18.6.763 1660448

[25] Matsusaka T Niimura F Shimizu A Pastan I Saito A Kobori H Liver angiotensinogen is the primary source of renal angiotensin II. J Am Soc Nephrol (2012) 23:1181–9 10.1681/ASN.2011121159 22518004 PMC3380650

[26] Gonzalez-Villalobos RA Janjoulia T Fletcher NK Giani JF Nguyen MT Riquier-Brison AD The absence of intrarenal ACE protects against hypertension. J Clin Invest (2013) 123:2011–23 10.1172/JCI65460 23619363 PMC3638907

[27] Campbell DJ Alexiou T Xiao HD Fuchs S McKinley MJ Corvol P Effect of reduced angiotensin-converting enzyme gene expression and angiotensin-converting enzyme inhibition on angiotensin and bradykinin peptide levels in mice. Hypertension (2004) 43:854–9 10.1161/01.HYP.0000119190.06968.f1 14769811

[28] Alexiou T Boon WM Denton DA Di Nicolantonio R Walker LL McKinley MJ Angiotensinogen and angiotensin converting enzyme gene copy number and angiotensin and bradykinin peptide levels in mice. J Hypertens (2005) 23:945–54 10.1097/01.hjh.0000166834.32817.41 15834279

[29] Textor SC Brunner HR Gavras H Converting enzyme inhibition during chronic angiotensin II infusion in rats. Evidence against a nonangiotensin mechanism. Hypertension (1981) 3:269–76 10.1161/01.HYP.3.2.269 6260647

[30] Mizelle HL Hall JE Woods LL Interactions between angiotensin II and renal nerves during chronic sodium deprivation. Am J Physiol Renal Physiol (1988) 255:F823–7 3056040 10.1152/ajprenal.1988.255.5.F823

[31] Shoback DM Williams GH Hollenberg NK Davies RO Moore TJ Dluhy RG Endogenous angiotensin II as a determinant of sodium-modulated changes in tissue responsiveness to angiotensin II in normal man. J Clin Endocrinol Metab (1983) 57:764–70 10.1210/jcem-57-4-764 6309884

[32] Koletsky RJ Gordon MB LeBoff MS Moore TJ Dluhy RG Hollenberg NK Captopril enhances vascular and adrenal responsiveness to angiotensin II in essential hypertension. Clin Sci (1984) 66:299–305 6362960 10.1042/cs0660299

[33] Hannedouche T Ikeni A Marques LP Natov S Dechaux M Schmitt F Renal effects of angiotensin II in normotensive subjects on short-term cilazapril treatment. J Cardiovasc Pharmacol (1992) 19(Suppl 6):S25–7 10.1097/00005344-199219006-00005 1382161

[34] Campbell DJ Do intravenous and subcutaneous angiotensin II administration increase blood pressure by different mechanisms? Clin Exp Pharmacol Physiol (2013) 40:560–70 10.1111/1440-1681.12085 23551142

[35] Campbell DJ Xiao H Fuchs S Bernstein KE Genetic models provide unique insight into angiotensin and bradykinin peptides in the extravascular compartment of the heart *in vivo*. Clin Exp Pharmacol Physiol (2009) 36:547–53 10.1111/j.1440-1681.2008.05106.x 19673938 PMC3142918

[36] Tian XL Pinto YM Costerousse O Franz WM Lippoldt A Hoffmann S Over-expression of angiotensin converting enzyme-1 augments cardiac hypertrophy in transgenic rats. Hum Mol Genet (2004) 13:1441–50 10.1093/hmg/ddh147 15128700

[37] Hagaman JR Moyer JS Bachman ES Sibony M Magyar PL Welch JE Angiotensin-converting enzyme and male fertility. Proc Natl Acad Sci U S A (1998) 95:2552–7 10.1073/pnas.95.5.2552 9482924 PMC19410

[38] Erdos EG Angiotensin I converting enzyme and the changes in our concepts through the years. Hypertension (1990) 16:363–70 10.1161/01.HYP.16.4.363 2170273

[39] Ganten D Hermann K Bayer C Unger T Lang RE Angiotensin synthesis in the brain and increased turnover in hypertensive rats. Science (1983) 221:869–71 10.1126/science.6879184 6879184

[40] Chappell MC Brosnihan KB Diz DI Ferrario CM Identification of angiotensin-(1-7) in rat brain. Evidence for differential processing of angiotensin peptides. J Biol Chem (1989) 264:16518–23 2777795

[41] Lawrence AC Clarke IJ Campbell DJ Angiotensin peptides in brain and pituitary of rat and sheep. J Neuroendocrinol (1992) 4:237–44 10.1111/j.1365-2826.1992.tb00165.x 21554603

[42] Senanayake PD Moriguchi A Kumagai H Ganten D Ferrario CM Brosnihan KB Increased expression of angiotensin peptides in the brain of transgenic hypertensive rats. Peptides (1994) 15:919–26 10.1016/0196-9781(94)90051-5 7984514

[43] Svensson P de Faire U Sleight P Yusuf S Östergren J Comparative effects of ramipril on ambulatory and office blood pressures. A HOPE substudy. Hypertension (2001) 38:e28–32 10.1161/hy1101.099502 11751742

[44] Ruiz-Ortega M Lorenzo O Ruperez M Esteban V Suzuki Y Mezzano S Role of the renin-angiotensin system in vascular diseases: expanding the field. Hypertension (2001) 38:1382–7 10.1161/hy1201.100589 11751722

[45] Dzau VJ Tissue angiotensin and pathobiology of vascular disease: a unifying hypothesis. Hypertension (2001) 37:1047–52 10.1161/01.HYP.37.4.1047 11304501

[46] Remuzzi G Perico N Macia M Ruggenenti P The role of renin-angiotensin-aldosterone system in the progression of chronic kidney disease. Kidney Int (2005) 68(Suppl 99):S57–65 10.1111/j.1523-1755.2005.09911.x 16336578

[47] Mann JF Schmieder RE McQueen M Dyal L Schumacher H Pogue J Renal outcomes with telmisartan, ramipril, or both, in people at high vascular risk (the ONTARGET study): a multicentre, randomised, double-blind, controlled trial. Lancet (2008) 372:547–53 10.1016/S0140-6736(08)61236-2 18707986

[48] Yusuf S Teo KK Pogue J Dyal L Copland I Schumacher H Telmisartan, ramipril, or both in patients at high risk for vascular events. N Engl J Med (2008) 358:1547–59 10.1056/NEJMoa0801317 18378520

[49] McMurray JJ Ostergren J Swedberg K Granger CB Held P Michelson EL Effects of candesartan in patients with chronic heart failure and reduced left-ventricular systolic function taking angiotensin-converting-enzyme inhibitors: the CHARM-Added trial. Lancet (2003) 362:767–71 10.1016/S0140-6736(03)14283-3 13678869

[50] Pfeffer MA McMurray JJ Velazquez EJ Rouleau JL Kober L Maggioni AP Valsartan, captopril, or both in myocardial infarction complicated by heart failure, left ventricular dysfunction, or both. N Engl J Med (2003) 349:1893–906 10.1056/NEJMoa032292 14610160

[51] Solomon SD Shin SH Shah A Skali H Desai A Kober L Effect of the direct renin inhibitor aliskiren on left ventricular remodelling following myocardial infarction with systolic dysfunction. Eur Heart J (2011) 32:1227–34 10.1093/eurheartj/ehq522 21317148

[52] Parving HH Brenner BM McMurray JJ de Zeeuw D Haffner SM Solomon SD Cardiorenal end points in a trial of aliskiren for type 2 diabetes. N Engl J Med (2012) 367:2204–13 10.1056/NEJMoa1208799 23121378

[53] Gheorghiade M Bohm M Greene SJ Fonarow GC Lewis EF Zannad F Effect of aliskiren on postdischarge mortality and heart failure readmissions among patients hospitalized for heart failure: the ASTRONAUT randomized trial. JAMA (2013) 309:1125–35 10.1001/jama.2013.1954 23478743

[54] Liu YH Yang XP Sharov VG Nass O Sabbah HN Peterson E Effects of angiotensin-converting enzyme inhibitors and angiotensin II type 1 receptor antagonists in rats with heart failure - Role of kinins and angiotensin II type 2 receptors. J Clin Invest (1997) 99:1926–35 10.1172/JCI119360 9109437 PMC508017

[55] Koid SS Ziogas J Campbell DJ Aliskiren reduces myocardial ischemia-reperfusion injury by a bradykinin B2 receptor- and angiotensin AT2 receptor-mediated mechanism. Hypertension (2014) 63:768–73 10.1161/HYPERTENSIONAHA.113.02902 24420538

[56] Ménard J Campbell DJ Azizi M Gonzales M-F Synergistic effects of ACE inhibition and Ang II antagonism on blood pressure, cardiac weight, and renin in spontaneously hypertensive rats. Circulation (1997) 96:3072–8 10.1161/01.CIR.96.9.3072 9386177

[57] Campbell DJ Kladis A Duncan A-M Effects of converting enzyme inhibitors on angiotensin and bradykinin peptides. Hypertension (1994) 23:439–49 10.1161/01.HYP.23.4.439 8144213

[58] Zeitz CJ Campbell DJ Horowitz JD Myocardial uptake and biochemical and hemodynamic effects of ACE inhibitors in humans. Hypertension (2003) 41:482–7 10.1161/01.HYP.0000054976.67487.08 12623947

[59] Linz W Wiemer G Gohlke P Unger T Schölkens BA Contribution of kinins to the cardiovascular actions of angiotensin-converting enzyme inhibitors. Pharmacol Rev (1995) 47:25–49 7784479

[60] Hornig B Kohler C Drexler H Role of bradykinin in mediating vascular effects of angiotensin-converting enzyme inhibitors in humans. Circulation (1997) 95:1115–8 10.1161/01.CIR.95.5.1115 9054837

[61] Gainer JV Morrow JD Lovelend A King DJ Brown NJ Effect of bradykinin-receptor blockade on the response to angiotensin-converting-enzyme inhibitor in normotensive and hypertensive subjects. N Engl J Med (1998) 339:1285–92 10.1056/NEJM199810293391804 9791144

[62] Campbell DJ Krum H Esler MD Losartan increases bradykinin levels in hypertensive humans. Circulation (2005) 111:315–20 10.1161/01.CIR.0000153269.07762.3B 15655136

[63] Campbell DJ Zhang Y Kelly DJ Gilbert RE McCarthy DJ Shi W Aliskiren increases bradykinin and tissue kallikrein mRNA levels in the heart. Clin Exp Pharmacol Physiol (2011) 38:623–31 10.1111/j.1440-1681.2011.05572.x 21736602

[64] Azizi M Chatellier G Guyene T-T Murieta-Geoffroy D Ménard J Additive effects of combined angiotensin-converting enzyme inhibition and angiotensin II antagonism on blood pressure and renin release in sodium-depleted normotensives. Circulation (1995) 92:825–34 10.1161/01.CIR.92.4.825 7641363

[65] Azizi M Menard J Bissery A Guyenne TT Bura-Riviere A Vaidyanathan S Pharmacologic demonstration of the synergistic effects of a combination of the renin inhibitor aliskiren and the AT1 receptor antagonist valsartan on the angiotensin II-renin feedback interruption. J Am Soc Nephrol (2004) 15:3126–33 10.1097/01.ASN.0000146686.35541.29 15579516

[66] Campbell DJ Anastasopoulos F Duncan A-M James GM Kladis A Briscoe TA Effects of neutral endopeptidase inhibition and combined angiotensin converting enzyme and neutral endopeptidase inhibition on angiotensin and bradykinin peptides in rats. J Pharmacol Exp Ther (1998) 287:567–77 9808682

[67] Richards AM Wittert GA Espiner EA Yandle TG Ikram H Frampton C Effect of inhibition of endopeptidase 24.11 on responses to angiotensin II in human volunteers. Circ Res (1992) 71:1501–7 10.1161/01.RES.71.6.1501 1423942

